# Sleeper Wire: A Case of Late Subcutaneous Migration of a Fractured Sternal Closure Cable 12 Years After Coronary Artery Bypass Graft

**DOI:** 10.7759/cureus.90915

**Published:** 2025-08-24

**Authors:** Benjoe George PS, Sabarish Nair, Subhash Chandra, Rohik Micka, Vasant PK

**Affiliations:** 1 Internal Medicine, Amrita Institute of Medical Sciences, Kochi, IND; 2 Emergency Medicine, Amrita Institute of Medical Sciences, Kochi, IND; 3 Cardiothoracic Surgery, Amrita Institute of Medical Sciences, Kochi, IND

**Keywords:** case report, chest x‑ray monitoring, coronary artery bypass grafting, late postoperative complication, sternal wire migration, subcutaneous plane

## Abstract

Sternal wire displacement is a rare but important postoperative complication following median sternotomy, commonly performed during cardiothoracic surgeries, such as coronary artery bypass grafting (CABG). While wire breakage and migration are known, migration into the deep subcutaneous plane without involvement of intravascular or muscular structures is extremely uncommon. We report a 71-year-old male with a history of triple vessel disease status post CABG 12 years prior, presenting with acute dyspnea. Imaging revealed pulmonary edema and an incidental finding of a migrated sternal wire in the deep subcutaneous plane anterior to the pectoralis major. Serial chest X-rays demonstrated gradual disruption and migration of the lowermost sternal wire over several years. The patient remains asymptomatic regarding the wire migration and is being managed conservatively with regular follow-up. This case highlights an unusual presentation of sternal wire migration confined to the subcutaneous plane, emphasizing the importance of long-term radiological surveillance in post-sternotomy patients. Although often asymptomatic, displaced sternal wires may pose significant risks, necessitating individualized management strategies. Conservative monitoring with periodic imaging is a reasonable approach when the patient is asymptomatic and the wire is stable.

## Introduction

Sternal wire displacement is a rare but clinically significant postoperative complication following median sternotomy, a common surgical approach in cardiothoracic procedures. Median sternotomy remains the standard surgical approach for most procedures requiring optimal exposure of the heart and mediastinal structures; however, new techniques for cardiac operations are being evaluated for their potential to reduce perioperative morbidity and accelerate postoperative recovery. Manubrium-sparing median sternotomy (MSS) is a uniform approach for cardiac operations. The initial experience with the MSS approach suggests several definite advantages. First, the incision is cosmetically acceptable to patients. At about 12 cm, the MSS incision is a little longer than the incisions made for other minimally invasive sternotomy techniques, but it is still much smaller than the incision for conventional median sternotomy. This is especially important for younger patients because they are often reluctant to have a scar near the neck [1]. Advances in surgical techniques and perioperative care have significantly reduced the incidence of related complications. However, serious postoperative events such as deep sternal wound infections (DSWIs) may continue to pose a significant clinical challenge, contributing to prolonged hospital stays and increased in-hospital mortality [2]. A rare but notable complication is sternal wire migration to unusual sites such as extra-sternal, intra-arterial, or even extra-thoracic locations, often with variable and sometimes serious clinical consequences [3,4-18]. Sternal dehiscence is frequently associated with mechanical failure of sternal wires, including breakage, displacement, or disruption, factors commonly identified in affected patients [19-22]. Sternal wire displacement is a highly specific sign for sternal dehiscence [23]. While fractures can happen at any time after surgery, wire migration is typically a late complication, usually occurring months to years following the procedure.

Here, we report a rare case of an incidentally detected migrated sternal wire positioned in the deep subcutaneous plane in a patient presenting with dyspnea.

## Case presentation

**Figure 1:**
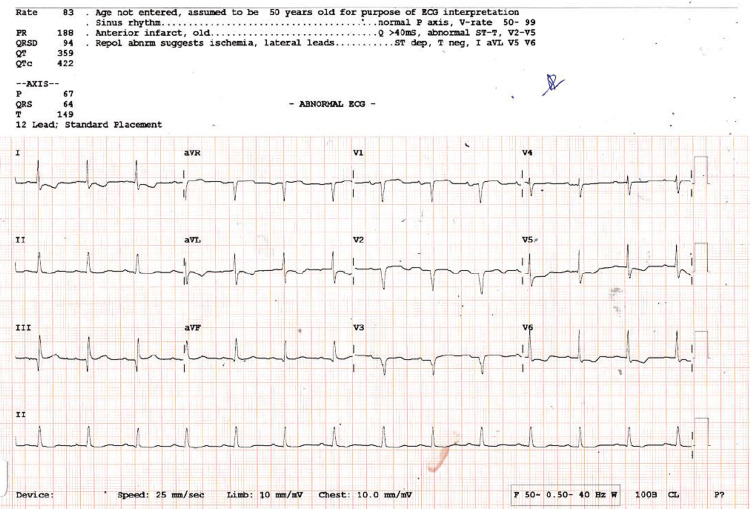
ECG showing new-onset T wave inversion in leads V5 and V6.

A 71-year-old male with a known history of triple-vessel coronary artery disease and prior coronary artery bypass grafting (CABG) performed 12 years ago, presented with a four-day history of acute shortness of breath. It was further aggravated on lying down and on exertion. There was a history of wheeze, orthopnea, and paroxysmal nocturnal dyspnea. It was associated with a non-productive cough and palpitations, with no accompanying fever or cyanosis. On examination, the patient was hypoxic, and the current X-ray revealed changes suggestive of pulmonary edema. The electrocardiogram (ECG) showed new-onset T wave inversion in leads V5-V6, as shown in Figure 1. The patient had undergone CABG 12 years ago. The immediate postoperative period was uneventful, and he has been on regular cardiology follow-up since then. The patient was a known case of type 2 diabetes mellitus, systemic hypertension, and dyslipidemia. He remained asymptomatic till recently, when he presented with the aforementioned symptoms.

**Figure 2:**
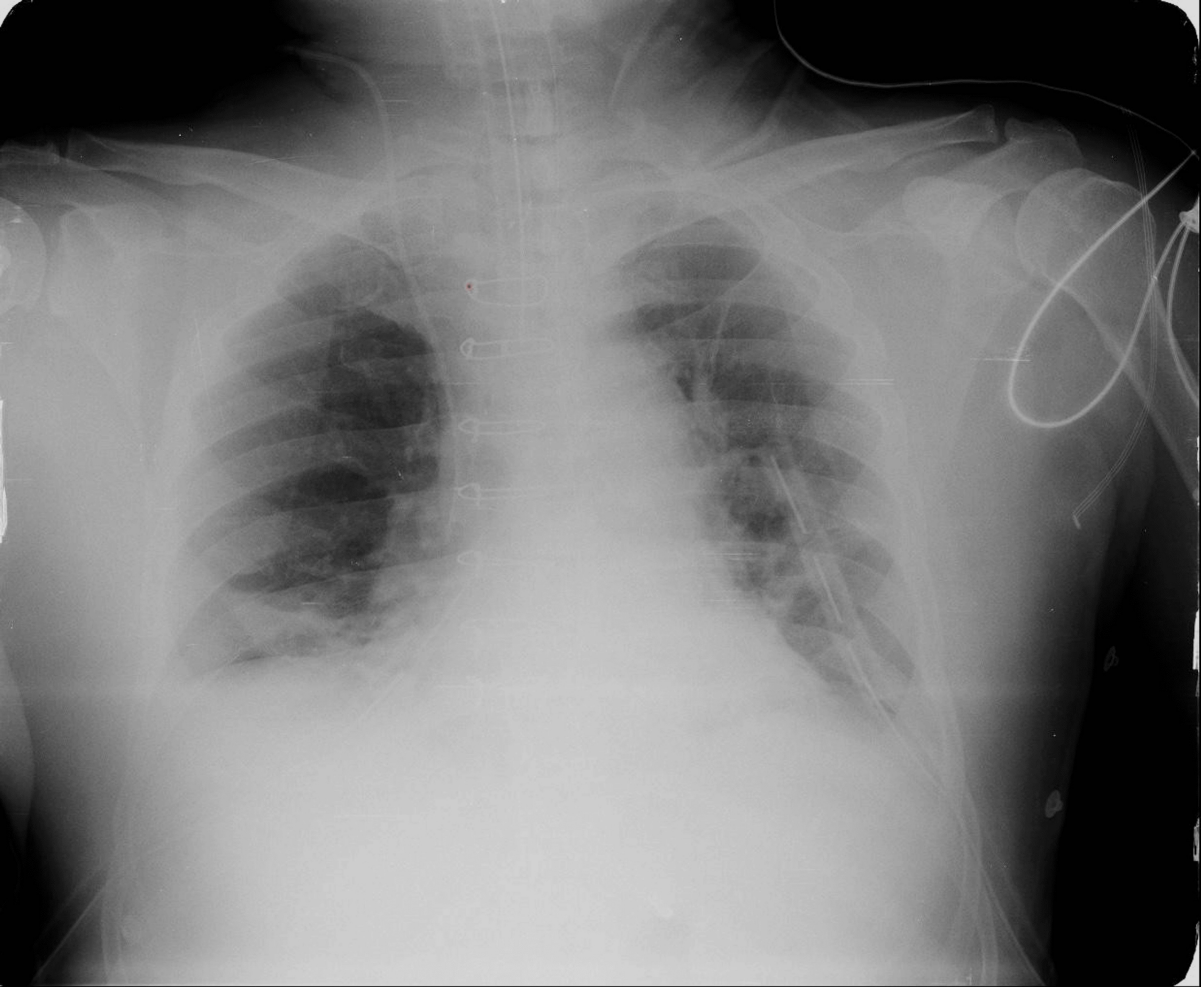
Chest X-ray post coronary artery bypass graft showing intact sternal wires (2013).

**Figure 3:**
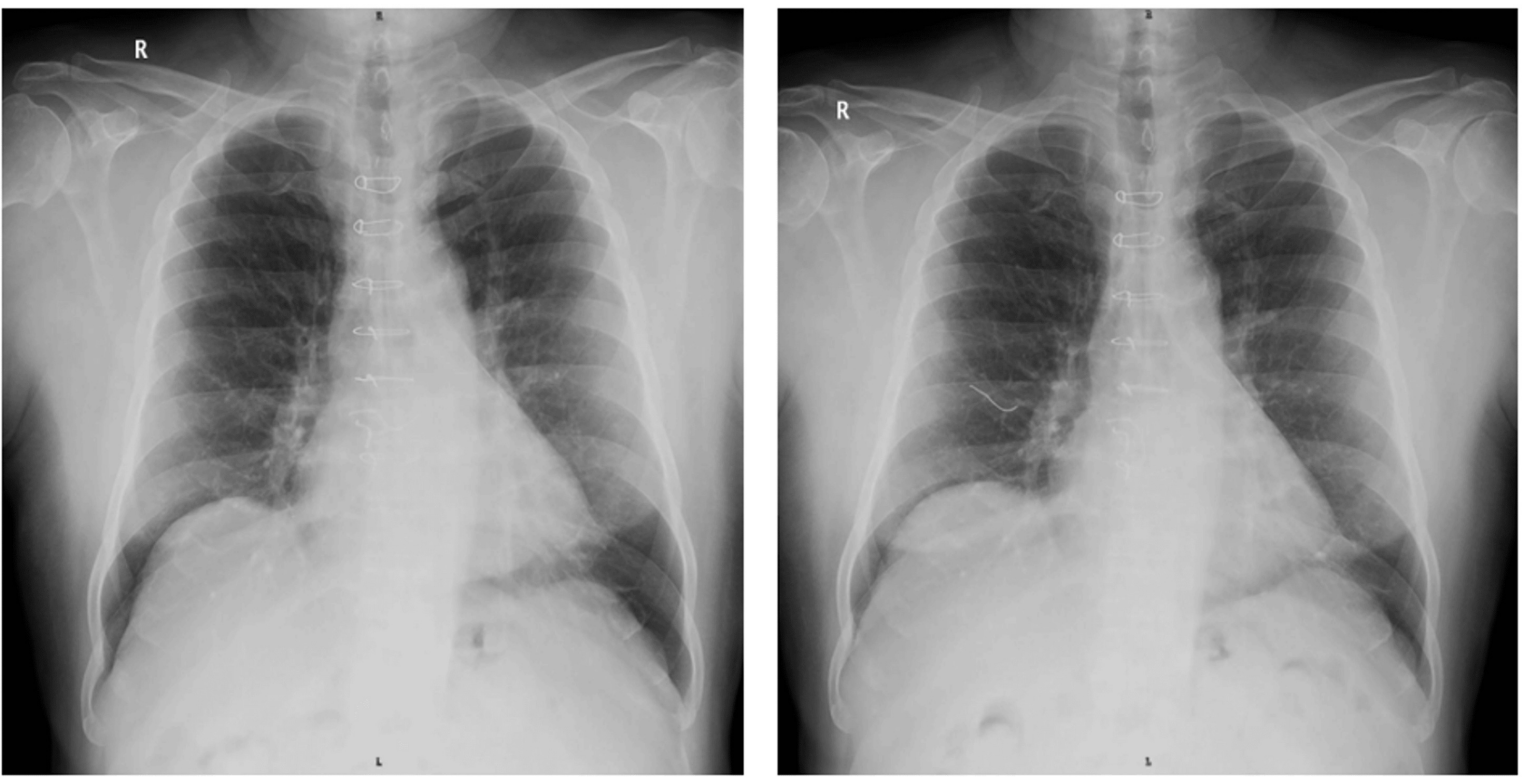
Lowermost sternal wire disruption and anterior migration four years post coronary artery bypass graft (2017).

As part of the diagnostic work-up for his acute respiratory symptoms, a chest X-ray was performed. Interestingly, it showed a wire-like radiopaque foreign body on the chest radiograph. This unexpected finding raised concerns and was further investigated by correlating it with a left lateral view X-ray of the chest to localize the structure and assess its significance. A review of serial X-rays taken over the past 12 years revealed some interesting findings. The chest X-ray done in the immediate postoperative period had revealed no significant changes, as shown in Figure 2. However, X-rays taken four years after surgery, during follow-up, showed that the lowermost sternal wire had broken and migrated, as shown in Figure 3 (wires had migrated toward the anterior mediastinum along the subcutaneous plane).

**Figure 4:**
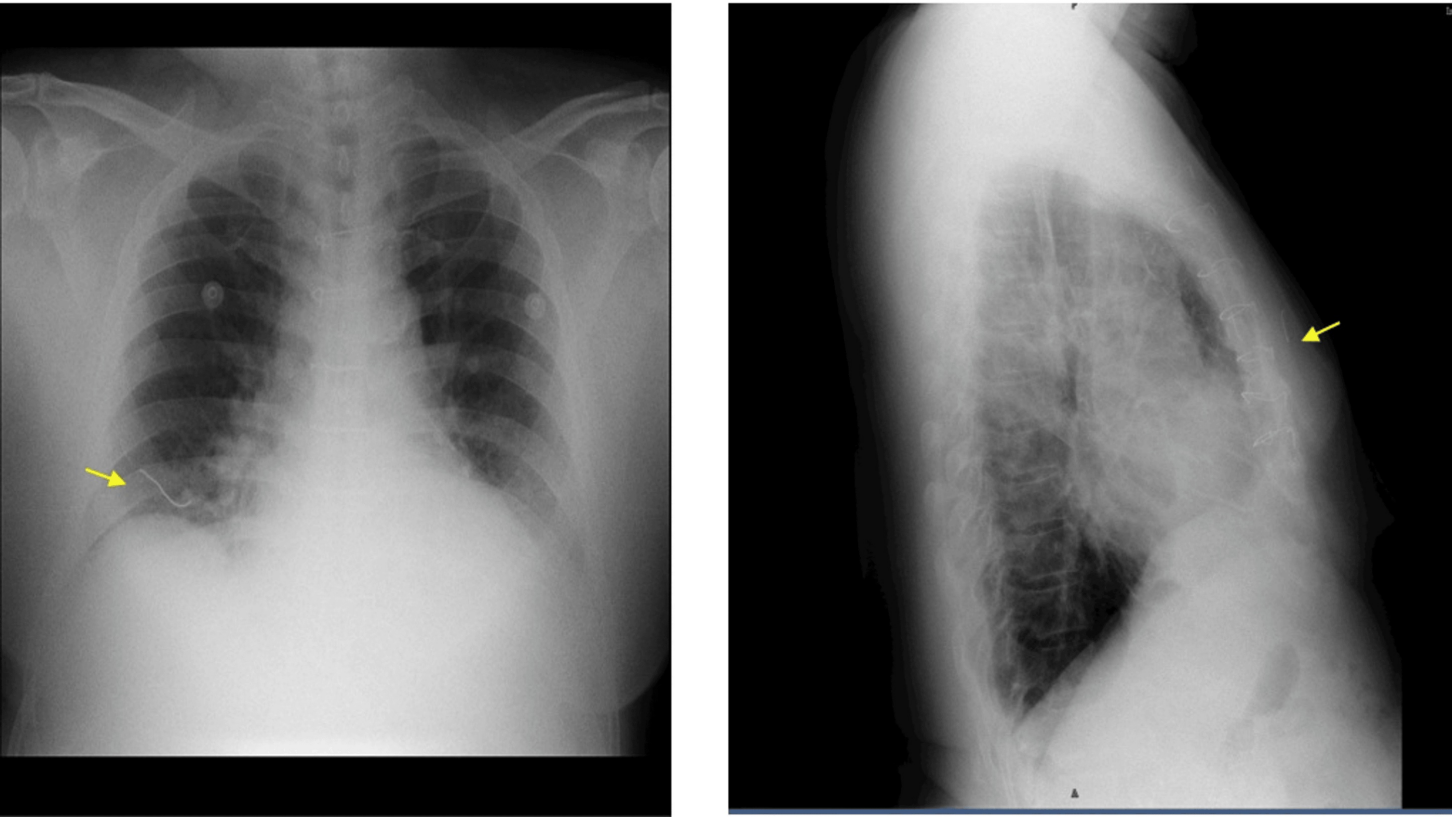
X-ray showing the sternal wire migrating along the anterior aspect of the right lung. Left lateral view showing the wire along the anterior mediastinal plane.

**Figure 5:**
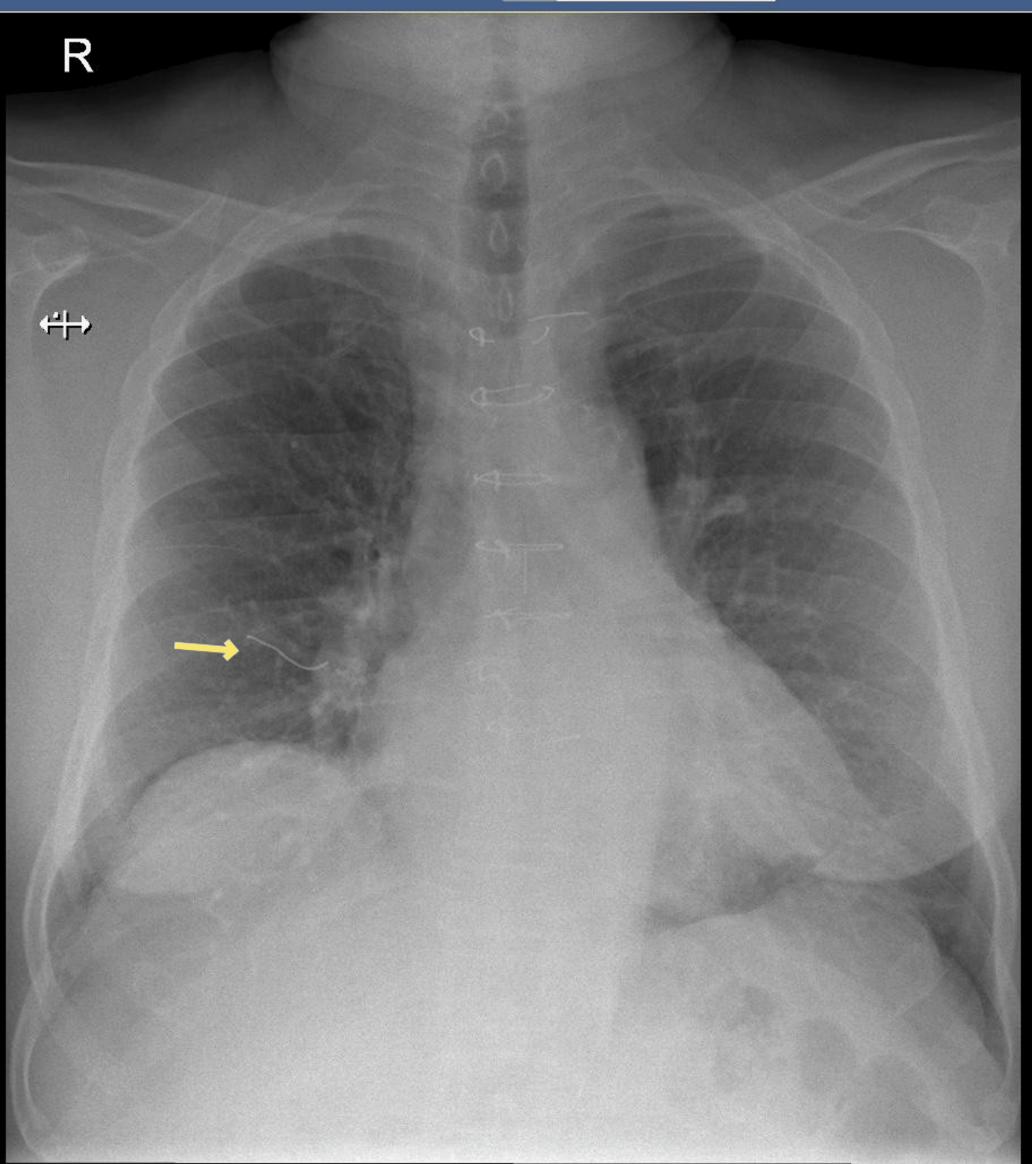
Chest X-ray showing the sternal wire migrating toward the anterior aspect of the right lung in the present day (2025).

**Figure 6:**
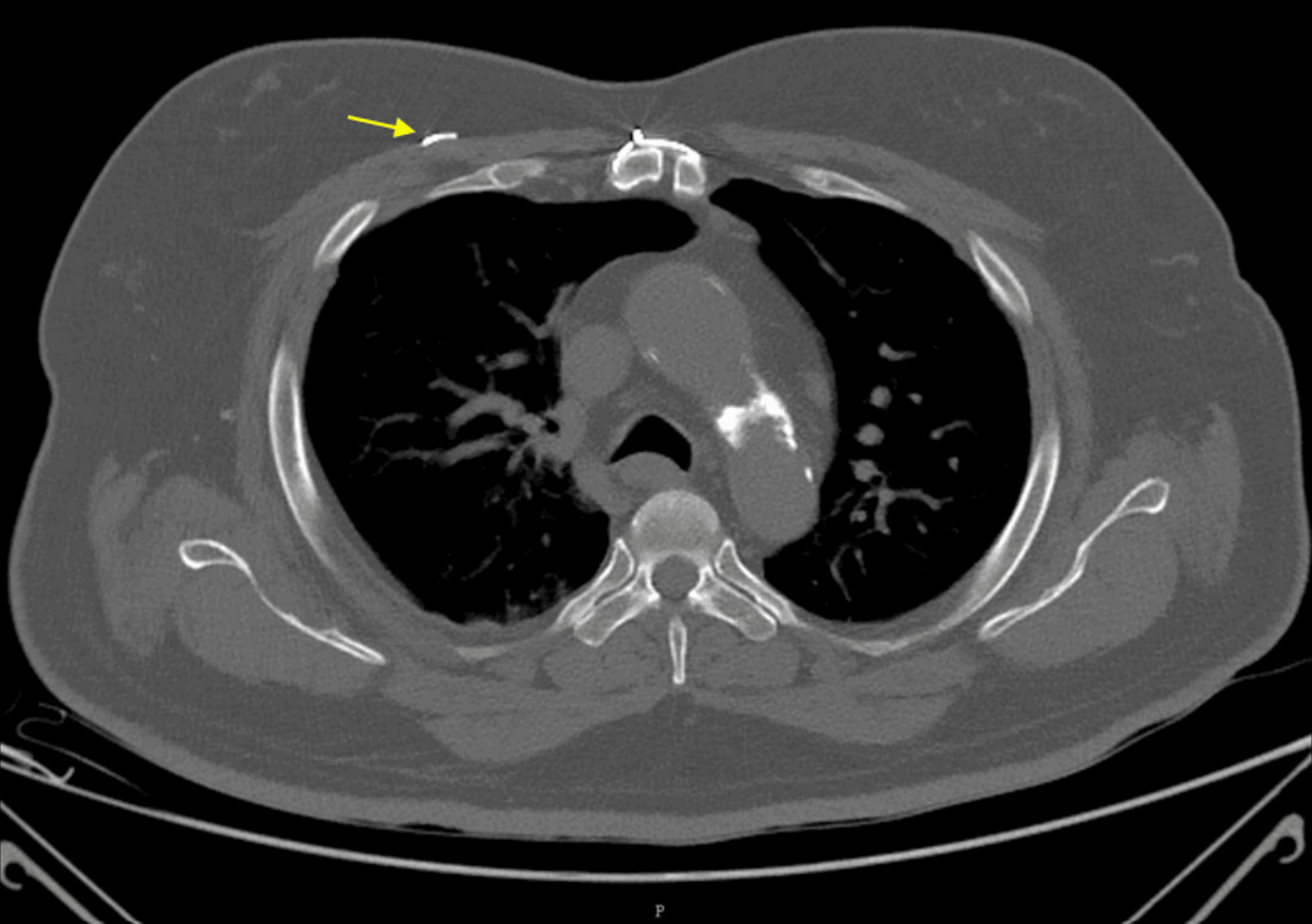
High-resolution computed tomography (HRCT) showing the sternal wire located in the deep subcutaneous plane just anterior to the pectoralis major.

This finding was not noted in earlier clinical assessments, possibly because the patient remained asymptomatic for several years. The wire in subsequent X-rays showed a change in its position, although its plane remained the same, as shown in Figures 4, 5. The continued positional change of the wire suggested progressive migration over time, potentially influenced by respiratory motion, musculoskeletal activity, or tissue remodeling. The plane of the wire being atypical, the exact anatomical location was determined after performing a high-resolution computed tomography (HRCT) scan, which revealed the wire to be located in the deep subcutaneous plane, anterior to the pectoralis major muscle, as demonstrated in Figure 6. The HRCT provided crucial detail that confirmed the wire had not penetrated deeper vital structures, but its proximity to major vessels and thoracic organs warranted close monitoring.

This case underlines a rare but important long-term complication following median sternotomy for sternal wire fracture and migration, which may go unnoticed unless patients become symptomatic or imaging is performed for unrelated reasons. While the current presentation was primarily due to acute decompensated heart failure, the radiographic finding of wire migration added a significant incidental dimension to the clinical picture and warranted further attention to exclude potential complications, such as wire erosion, vascular injury, or infection.

As the patient is currently asymptomatic, a plan was made after consultation with a cardiovascular thoracic surgeon (CVTS) to keep him on six-month follow-ups.

## Discussion

To the best of our knowledge, this is the first report of a sternal wire migrating exclusively within the subcutaneous plane without involving intravascular or intramuscular structures, as shown in Figure 6. Although not life-threatening, wire removal was deferred in favor of conservative monitoring.

Sternal wound complications contribute to morbidity and mortality post cardiac surgery, with sternal wound dehiscence in approximately 5% of cases [4]. Prompt recognition is key. Malalignment impairs healing, and fragments may puncture vital structures, making dehiscence a surgical emergency. Chronic post-sternotomy pain affects up to 50% of patients, with severe pain in 5-10% [23]. Causes include nerve injury, instability, or wire irritation. Sternal wire removal can offer relief in selected patients where the sternum has fully healed. Conservative treatment with nonsteroidal anti-inflammatory drugs (NSAIDs) remains the first-line treatment [24].

Patient-related risk factors include diabetes mellitus, obesity, chronic obstructive pulmonary disease (COPD), poor nutrition, and respiratory distress. Postoperative infections and physical strain increase risks. Similarly, the number of sternal wires and fixation pairs could have been a contributing factor. There is an increased incidence of sternal complications in patients with comorbidities like chronic obstructive pulmonary disease and diabetes mellitus, especially when using seven or fewer sternal wires. Technical failures include improper wire placement, inadequate quantity, poor technique, or bone fragility. Understanding and addressing these factors is essential to reducing the risk of this complication. In most cases of sternal wire fracture, migration, or displacement, diagnosis is typically made via chest X-ray, with a CT scan reserved for atypical locations. Sternal wire migration is generally managed conservatively unless complications arise.

Reported wire migration complications include cardiac tamponade [14], aortic injury [10], hemoptysis [12], and endocarditis [24]. Boiselle et al. found lateral wire displacement observed in 84% of patients, and wire disruption was found in 21% of dehisced wounds [9]. Wires have migrated up to 25 cm, including to the posterior-lateral abdominal wall [13]. Closure techniques like Robicsek's double-wiring stabilize high-risk patients like those with obesity or COPD. Sternal plate fixation offers secure closure with less postoperative pain and narcotic use, though hospital stays are similar [23].

The location of the sternal wire plays a crucial role in the management of the case. Intravascular or intramuscular migration requires prompt surgical intervention, whereas a non-invasive wire without significant complaints requires close surveillance.

## Conclusions

Sternotomy wire migration is an uncommon occurrence, but should it occur, the consequences may be potentially fatal, resulting in embolization to cardiac tamponade.

The progressive wire fracture and migration seen in our patient were likely attributable to ongoing sternal motion caused by sternal non-union, which was further exacerbated by the patient’s chronic breathlessness. Contributing to the increased risk of sternal wound complications were several additional comorbidities, including diabetes mellitus, obesity, bronchial asthma, and the number of sternal wires. In patients with sternal wire migration, the wires typically remain intact, distinguishing this complication from cases involving wire fracture. Instead of mechanical breakage, migration results from repetitive lateral stress caused by chest wall motion, particularly during respiration or patient repositioning in the early postoperative period. This tension can lead the wires to gradually erode through or pull across the sternal bone, even when the bone remains structurally intact. Over time, this process may result in significant displacement of the wire, often without immediate clinical symptoms. The presence of a displaced wire fracture or a potentially migratory segment constitutes a strong indication for operative repair. If conservative management is chosen, close surveillance is mandatory, and any changes in wire integrity must be meticulously assessed on each chest roentgenogram.
